# A Universal Intelligent System-on-Chip Based Sensor Interface

**DOI:** 10.3390/s100807716

**Published:** 2010-08-17

**Authors:** Virgilio Mattoli, Alessio Mondini, Barbara Mazzolai, Gabriele Ferri, Paolo Dario

**Affiliations:** 1 Istituto Italiano di Tecnologia (IIT), Center for Micro-BioRobotics IIT@SSSA, Viale Rinaldo Piaggio, 34, 56025 Pontedera (PI), Italy; E-Mails: barbara.mazzolai@iit.it (B.M.); dario@sssup.it (P.D.); 2 Scuola Superiore Sant’Anna, piazza Martiri della Libertà, 33, 56127 Pisa, Italy; E-Mails: alessio.mondini@forsense-tech.com (A.M.); g.ferri@sssup.it (G.F.)

**Keywords:** smart sensors, configurable interface, IEEE 1451, monitoring systems

## Abstract

The need for real-time/reliable/low-maintenance distributed monitoring systems, e.g., wireless sensor networks, has been becoming more and more evident in many applications in the environmental, agro-alimentary, medical, and industrial fields. The growing interest in technologies related to sensors is an important indicator of these new needs. The design and the realization of complex and/or distributed monitoring systems is often difficult due to the multitude of different electronic interfaces presented by the sensors available on the market. To address these issues the authors propose the concept of a Universal Intelligent Sensor Interface (UISI), a new low-cost system based on a single commercial chip able to convert a generic transducer into an intelligent sensor with multiple standardized interfaces. The device presented offers a flexible analog and/or digital front-end, able to interface different transducer typologies (such as conditioned, unconditioned, resistive, current output, capacitive and digital transducers). The device also provides enhanced processing and storage capabilities, as well as a configurable multi-standard output interface (including plug-and-play interface based on IEEE 1451.3). In this work the general concept of UISI and the design of reconfigurable hardware are presented, together with experimental test results validating the proposed device.

## 1. Introduction

The need for real time, reliable, low maintenance distributed monitoring systems is becoming nowadays more and more obvious in several applications in the environmental, agro-food, medical, and industrial fields [1–3]. In this sense the growing interest in sensor network technologies and in sensor related technologies in general, is an important indicator of these new needs.

Numerous transducers are available on the market with a multitude of different interfaces to provide measurements of all kinds. Typical front-ends are voltage output, current output, capacitive or resistive outputs, and several digital interfaces such as RS232, I^2^C, SPI, frequency and bus based interfaces. The presence of such a large number of different interfaces often makes the design and the realization of complex and/or distributed monitoring systems complex [4]. Moreover, in order to design reliable and effective distributed monitoring systems, other fundamental aspects must be taken into account, such as the performance and the reliability of the sensors used for the system implementation.

To address these issues the concept of an intelligent sensor (also referred to as a smart sensor) was introduced in the recent past [5,6]. A smart sensor can be defined as a sensor with some kind of embedded intelligence (usually provided by a microcontroller), able to carry out advanced functions such as embedded signal conditioning, self-calibration, self-identification, diagnostic and networking activities [7–10]. In order to achieve these advanced functionalities several smart interfaces for transducers have been recently proposed, often based on ASICs solutions [11,12].

Some commercial sensors show different degrees of “smartness”. However the standardization of the interfaces is still an open issue. An important effort in this direction was the introduction of the IEEE 1451 [13–19] standard for intelligent sensors. This standard proposes functionalities, data structures and communication protocols aimed at making possible transducer-to-network inter-changeability and transducer-to-network interoperability [20], thus simplifying the implementation (realization) of complex distributed monitoring systems.

Nevertheless, at present, examples of intelligent sensors available on the market and compliant with this standard are still limited [21]. To solve this problem, some dedicated hardware interfaces based on the IEEE 1451 standard, able to interface with different sensor typologies were recently proposed. These proposed devices are usually based on relatively complex dedicated electronic boards [22–30].

With this in mind, the authors propose a new low-cost system to convert a generic transducer into a intelligent sensor with multiple standardized wired interfaces. This innovative system is called Universal Intelligent Sensor Interface (UISI). It provides a flexible analog and/or digital front-end (including conditioning and conversion functions), able to interface different transducer typologies, while providing enhanced processing and storage capabilities and a configurable multi-standard output interface (including plug-and-play interface inspired to IEEE 1451.3 standard). A similar approach based on reconfigurable FPGA (Field Programmable Gate Array) and FPAA (Analog Array), compliant with IEEE 1451.4 standard, have been also very recently proposed [31].

The presented work is structured as follows: in the first part the general concept of the UISI is presented. Then, the design and implementation section describes the hardware board realization, the dynamic analog/digital front-end configuration, and the firmware/software development. Experimental characterization results tests, in the lab and in real applications, are then presented and discussed.

## 2. Universal Intelligent Sensor Interface Concept and the IEEE 1451 Standard

**Figure 1.**
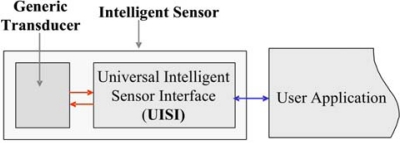
Schematic diagram of the Universal Intelligent Sensor Interface (UISI) concept: the UISI converts a generic transducer into an intelligent sensor.

The Universal Intelligent Sensor Interface (UISI) intends to provide a quick and reliable solution to convert a common generic transducer into a intelligent sensor with plug and play features (Figure 1).

**Figure 2.**
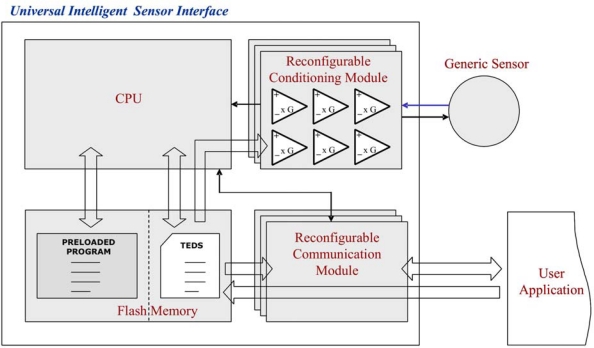
Architecture of UISI.

The UISI achieves this goal by providing a firmware configurable analog front-end circuit, some computational capabilities, a memory for data and for configuration parameters, and one or more standardized output connections. Figure 2 shows the architecture of the proposed device.

The core of the UISI interface is a reconfigurable conditioning module, composed by several operational amplifiers (with selectable gains) and digital modules that can be connected each other via firmware in different ways, providing the required complete front-end for different types of sensors, including single/differential amplification, analog to digital conversion, powering and filtering. The generic sensor is connected to this conditioning module directly through a 4-line connection (two lines for power supply and up to two lines for signals). Then, in some configurations an additional resistor must be added.

The conditioning module is directly configured by the CPU block that also manages the other functionalities of the device. The CPU configures the conditioning module at the power-up time by using the configuration data stored in a non-volatile memory. The data necessary for the plug and play functionality are stored in this memory in the form of a Transducer Electronic Datasheet (TEDS) [18] following the philosophy of the IEEE 1451 standard. The TEDS contains an electronic description of the board and sensors (transducer channels) connected. The board is described by the META TEDS that provides information as the Universal Unique ID for unequivocal identification and the number of connected transducer channels. The Transducer channel TEDS contains information about the sensor connected and in particular maximum and minimum values, physical unit, acquisition time, warm up time, and more. A textual description for board and sensors is also supplied.

The CPU manages the signal/measurement acquisition from the sensor, through the conditioning module, executing the program stored in another area of the non-volatile memory, and using the parameters contained in the TEDS (type of data, acquisition time, update rate, calibration parameters, *etc.*). The acquired data are buffered in a volatile memory and then sent outside through customizable interfaces. A standard 8-bit CPU core is suitable for the application, due to the relatively low computational performance required by the system. The main communication interface is a custom bus-type interface inspired by the IEEE 1451.3 standard [16], connecting the UISI device to a master external device, like a Network Capable Application Processor (NCAP in IEEE 1451).

Through this interface, data and commands are exchanged between the UISI device and the NCAP; providing the possibility of TEDS handling and of a plug-and-play connection. TEDS and configuration parameters for the conditioning module can be uploaded at run-time into the UISI device through this interface. Moreover the device supports other types of external connections. Like the conditioning module, the communication module can also be reconfigured by using the stored configuration parameters, to support simpler and more direct output formats, in order to make the use of the device more flexible. This aim was achieved adding a 0–5 V analog output and a RS232 digital output as communication interfaces.

All the UISIs are programmed with the same software, which allows the management of every configuration and the communication with the NCAP. In a second phase, when a certain UISI must be connected to a specific sensor, the TEDS are created and loaded on the UISI. Five kinds of TEDS are stored on the UISI when it is configured:

- META TEDS: contains a parametric description of the UISI content;
- META TEXT TEDS: contains a textual description of the UISI content;
- TC TEDS: one for channel, contains a parametric description of the sensor (or actuator) connected;
- TC TEXT TEDS: one for channel, contains a textual description of the connected sensor;
- CONFIG TEDS: contains the HW parameters for the UISI configuration for a specific sensor.

**Figure 3.**
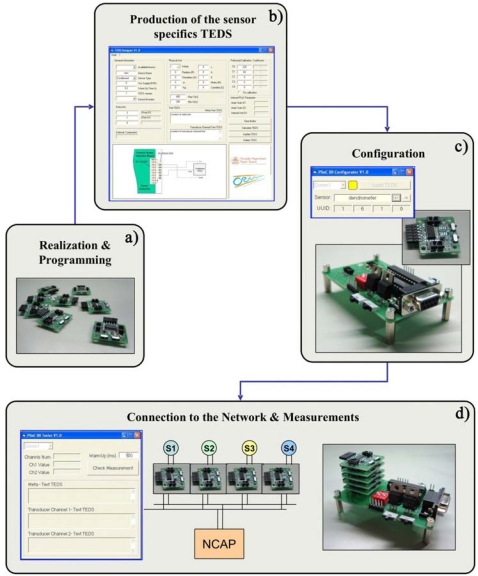
UISI programming cycle: **(a)** programming of all the UISIs with a common firmware that contains all the required functionalities; **(b)** TEDS is generated on the base of the specific sensor to be integrated; **(c)** TEDS parameters are loaded on the board by configuration tools; **(d)** UISI is connected to the sensor and to the NCAP and is ready for working.

The first four kinds of TEDS are sent by the UISI to the NCAP during the plug & play service, in order to describe a new intelligent sensor to the network. The CONFIG TEDS is normally managed by a specific software suite (configurator tools). Figure 3 shows the whole chain involved in the UISI programming.

For the management of the UISIs along the whole chain, three software tools and a configurator device (Configuration Downloader) have been developed. The configurator device is essentially a NCAP simulator. It integrates the whole NCAP functionalities and it communicates with a PC through an RS232 connection. The three developed tools are:

- TEDS Designer software devoted to create suitable TEDS for the specific sensors. The software allows a simple customization of the UISI, automatically generating a TEDS that describes the sensor and storing it in a dedicated database, through the input by the user of several parameters about the specifications of the sensor.
- UISI Configurator software devoted to downloading the TEDS from the database to the UISI. To perform the upload operations on the UISI, it is necessary to use the Configuration Downloader, connected to the PC by a RS232 serial connection.
- UISI Tester software visualizes the measurements sent by the UISI on the PC screen, using the Configuration Downloader as interface.

The configuration of the UISI permits to transform a generic sensor in a intelligent sensor with plug & play capability (see Figure 1). All the information useful to correctly describe the sensor is inserted in the UISI memory, structured as the TEDS.

## 3. Universal Intelligent Sensor Interface Design

### 3.1. Hardware Board Design

The specific requirements of UISI devices in terms of flexibility, adaptability and functionalities can be achieved by using different technological solutions, depending on the expected performances and the costs. Two standard implementable solutions are the following: 1) the development of a digital/analog mixed ASIC with configuration capability; 2) the development of a relatively complex electronic board including a microcontroller, operational amplifiers, programmable gain amplifiers (PGA), Analog to Digital Converters (ADC), Digital to Analog Converters (DAC) and analog switches. Both solutions have several drawbacks. In particular, the first solution presents some limits: inflexible design, the deployed systems are not upgradable, difficulties for rapid time-to-market designs and the need of complex and expensive development tools. On the other hand, the second solution is not reprogrammable at run-time, and it typically has high production costs and relatively large size. For these reasons, this work presents a different solution, based on a commercial available multifunctional chip, which allows overcoming such drawbacks. A Programmable System on Chip (PSoC) produced by Cypress [32] is used to implement the UISI device. The use of these devices makes possible the connection of analog and digital output sensors through the implementation of a TEDS, an intelligent power management (based on programmable sensors power supply handling and microprocessor sleep mode) and the possibility of providing multi-standard external connections.

The PSoC™ family consists of many Mixed Signal Arrays with On-Chip Controller devices. These devices are designed to replace traditional systems based on multiple analog/digital components with one, low cost, programmable single-chip component. A PSoC device includes configurable blocks of analog and digital logic, as well as programmable interconnections (all reconfigurable at run-time). This architecture allows the creation of customized peripheral configurations, to match the requirements of each individual application. Additionally, a fast 8 bit CPU, Flash program memory, SRAM data memory, and configurable IO are included. The PSoC device used is the CY8C29466 model. The PSoC features make the implementation of UISI device possible in a single-chip: the internal analog and digital logic blocks are used to generate at run-time the front-end suitable for the generic transducer to be interfaced. The information for the front-end configuration is contained as a TEDS in the built-in flash memory, which can be re-written at run-time. The PSoC also provides resources to implement the communication with the NCAP or through auxiliary outputs.

The UISI device was built by integrating the PSoC chip in a small PCB board with two connectors. One connector is used to interface the selected transducer (sensor); the other one supplies power and connectivity to the whole device. The transducer interface requires only six lines:

- two (or one) lines for the sensor signal (Gsens+, Gsens−);
- two lines for an external resistor necessary only in some configurations (R_0_′, R_0_″) that can be also mounted directly on the UISI;
- two lines for the power supply of the sensors (Vpp-sens, Gnd).

**Figure 4.**
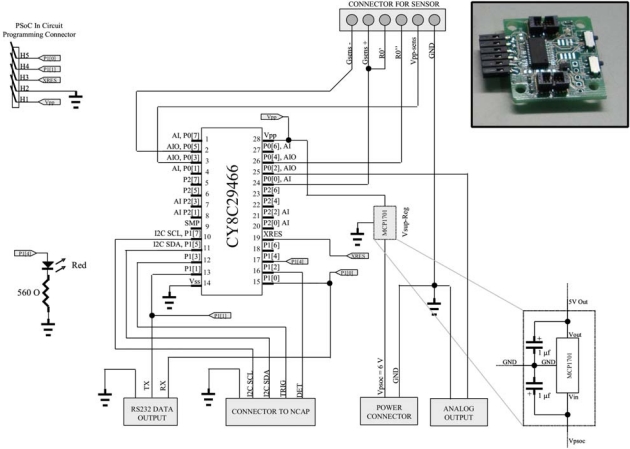
Schematic and the picture of the implemented UISI interface.

The functionality of such lines depends on the sensor type (and thus on the internal configuration of the PSoC chip). In the next section all different supported configurations will be examined into detail, including the sensor interface pin-out. Figure 4 shows the schematics and a picture of the implemented UISI interface.

### 3.2. Reconfigurable Analog/Digital Modules Design

In order to interface the selected transducer in the proper way, the PSoC device has to configure the analog and digital blocks obtaining the suitable front-end architecture, based on the information contained in a custom TEDS (Configuration TEDS) stored in the flash memory. The supported hardware configurations are implemented at design time and can be switched at run-time. The Configuration TEDS contains the parameters necessary to select and to tune the required hardware configuration. Up to now, front-ends for eight common sensor typologies were implemented, in particular:

- Conditioned Sensor—Configuration 1;
- Unconditioned Sensor (Wheatstone bridge like)—Configuration 2;
- Current Output Sensor—Configuration 3;
- Resistive Sensor—Configuration 4;
- Capacitive Sensor—Configuration 5;
- Digital Sensor (Serial interface type: RS232/SPI/Microwire)—Configuration 6/7/8

The maximum available voltage for the sensor supply is 5 V in all the supported configurations, and the maximum sensor sourcing current is 30 mA, with the power being supplied directly by the PSoC chip by using an internally buffered DAC (with some limitations arising from the PSoC chip manufacturer’s specifications).

For each type of sensor a specific architecture is required. These architectures are implemented at the firmware level by configuring the reconfigurable analog/digital blocks. Several implementations in the PSoC device will be discussed in the following sections. In particular, for each configuration, we will explain the general logical block scheme and the implementation made by using the Cypress PSoC Designer tools.

#### 3.2.1. Conditioned Sensor—Configuration 1

The output for a conditioned sensor is usually 0–5 V (or similar). The output impedance is relatively low, and thus the signal does not require any amplification. Moreover, the output of the sensor is referred to the ground (no differential input required).

**Figure 5.**
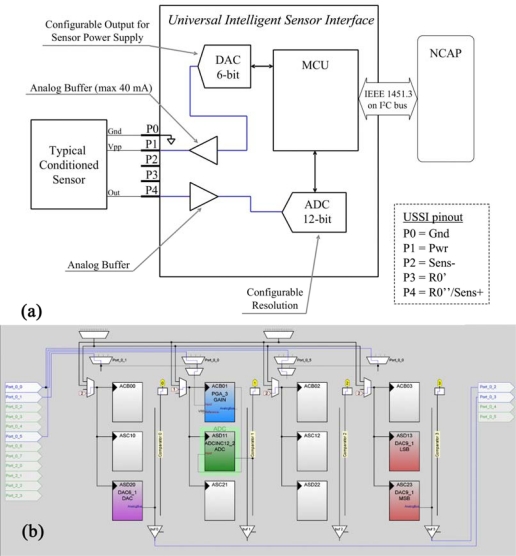
Conditioned Sensors: **(a)** Logical scheme of UISI conditioning front-end and **(b)** screen-shot of related implementation in PSoC device through by means of Graphic Configurator.

The conditioning scheme [shown in Figure 5(a)] is consequently relatively simple: the acquisition module is composed by an input buffer connected to an ADC (with selectable 8–14 bit resolution). The power for the sensor is supplied by an internal buffered 6-bit DAC.

Figure 5(b) shows the implementation of the analog part of this configuration, carried out by means of Graphic Configurator (included in the PSoC Designer tools, Cypress US). In this configuration the first block is the DAC6_1, used for generating the power supply of the sensor. It is a 6-bit DAC and guarantees a resolution of about 80 mV. The DAC6_1 output is connected to PORT_0_3 of the PSoC that is physically connected to the output connector and in particular to the Vpp-sens pin. An external power supply can be used when the 5 V voltage is too low or the sensor needs a more stable power supply (the DAC output is rather noisy). The PGA_3 is used as an analog input buffer for the sensor signal. The output of the sensor is connected to PORT_0_0 and then with the input of the PGA_3. In this configuration (for conditioned 0–5 V sensors) the gain of PGA_3 is normally set at a value of 1, but for other sensors that do not exploit the whole 0–5 V dynamics a proper gain can be introduced. The ADCINC12 is an incremental ADC with 12 bit resolution. DAC9_1 is a 9-bit DAC and is used for the power supply of the analog output proportional to the sensor output. It is connected to PORT_0_2 and then to the analog output connector. It is present in all the configurations.

#### 3.2.2. Unconditioned Sensor (Wheatstone Bridge Like)—Configuration 2

**Figure 6.**
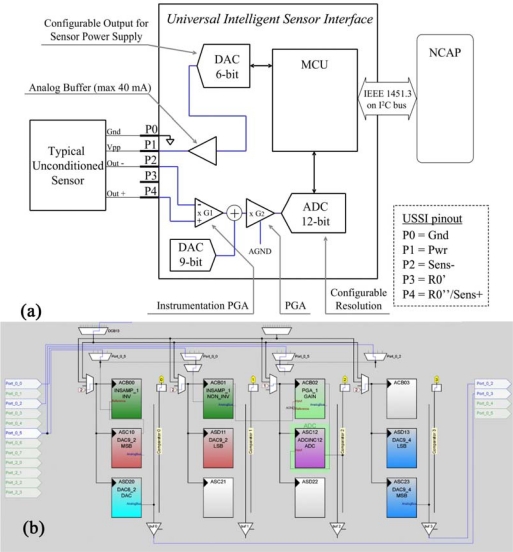
**(a)** Logical scheme of UISI conditioning front-end and **(b)** screen-shot of related implementation in PSoC device through by means of Graphic Configurator.

The differential output of an unconditioned sensor presents typically a very low voltage difference (in the range of mV). The output impedance is relatively high and thus the signal requires an amplification stage. Furthermore the signal common mode is likely different from the UISI ground, making a differential amplification necessary. The conditioning scheme for unconditioned (Wheatstone bridge like) sensor is shown in Figure 6(a).

The first stage of the acquisition module is composed by a programmable gain instrumentation amplifier with gain (G1) fixed to ×10. After this pre-amplification stage, a second amplification stage with offset compensation is implemented. The offset compensation, carried out by a 9 bit DAC, is required in the case of sensors with a large initial offset (on the order of the full span of the signal). The second amplification stage is composed by a Programmable Gain Amplifier (PGA) (gain = G2, 1 < G2 < 48). Both the offset compensation and the gain are automatically selected by firmware on the basis of the configuration parameters loaded in the TEDS. After the amplification stages, an ADC (with selectable 8–14 bit resolution is available for the acquisition. The power for the sensor is supplied by an internal buffered 6-bit DAC. Figure 6(b) shows the implementation of the analog part of this configuration, carried out using Graphic Configurator.

In this configuration, the first block is also a DAC6. It is used for generating the power supply of the sensor, with the same considerations as for the Configuration1. The sensor output is connected to the PORT_0_0 (Sens+) and PORT_0_5 (Sens−) and the signal is amplified by a (INSAMP_1) amplifier, which also corrects the offset of the signal adding the voltage supplied by the DAC9_2:

(1) $$V_{0-\mathrm{INSAMP}}=\left(V_{\mathrm{IN}+}-V_{\mathrm{IN}-}\right)\times G_{1}+V_{\mathrm{DAC}9}$$

At the end of this first stage of amplification, the signal is amplified and translated at a value close to AGND, that is the Analog Ground of the PSoC, and corresponds to Vcc/2. The second stage of amplification is composed by the PGA_1 with a reference voltage of AGND. In the end the amplified signal is acquired by the ADCINC12. DAC9_4 is the DAC for the analog output:

(2) $$V_{\mathrm{ADC}}=\left(V_{0-\mathrm{INSAMP}}-V_{\mathrm{AGND}}\right)\times G_{2}+V_{\mathrm{AGND}}$$

#### 3.2.3. Current Output Sensor—Configuration 3

**Figure 7.**
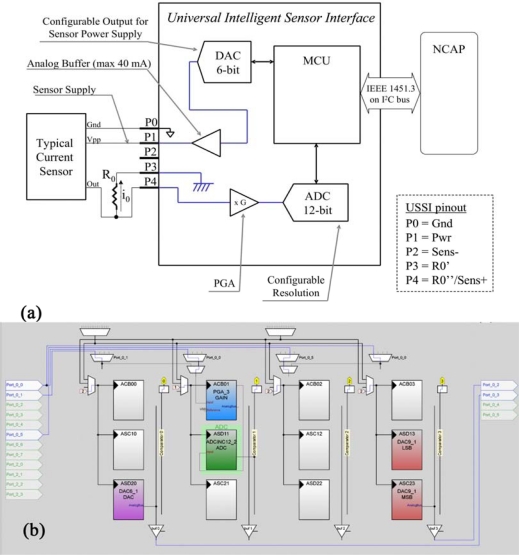
Current Output Sensors: **(a)** Logical scheme of UISI conditioning front-end and **(b)** screen-shot of related implementation in PSoC device through by means of Graphic Configurator.

In the case of a Current Output sensor an external precision resistor is required in order to perform the conditioning (the resistor is external to the PSoC, but it is included in the UISI device, and cannot be changed by configuration parameters). Figure 7(a) shows the conditioning scheme for this configuration. The current output of the sensor passes through the reference resistor connected to the UISI ground; in this way, the current is converted to a voltage.

The voltage drop across the resistor is amplified by a programmable gain amplifier: the gain (G) is selected by firmware based on the configuration parameters loaded in the TEDS (from G = x1 to G = x93). After the amplification stage an ADC (with selectable resolution: 8–14 bit) carries out the digitalization. The power for the sensor is supplied by an internal buffered 6-bit DAC.

Figure 7(b) shows the implementation of the analog part of this configuration, carried out using Graphic Configurator. This configuration has the same components and connections of conditioned sensor configuration. The only differences are that the PGA is used as an analog input buffer (unitary gain), as well as a real amplifier to adjust the input signal in the ADC range. Moreover, the PORT_0_4 (R0’) is put to ground via firmware. DAC9_1 is the DAC for the analog output:

(3) $$V_{\mathrm{ADC}}=I_{\mathrm{Sensor}}\times R_{0}\times G$$

#### 3.2.4. Resistive Sensor—Configuration 4

**Figure 8.**
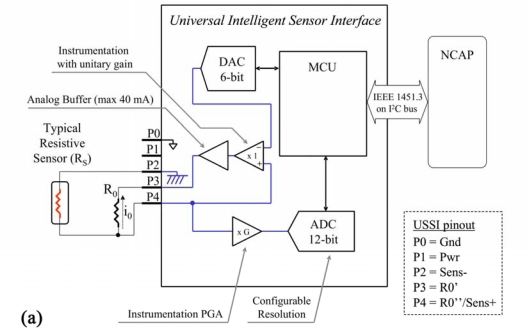

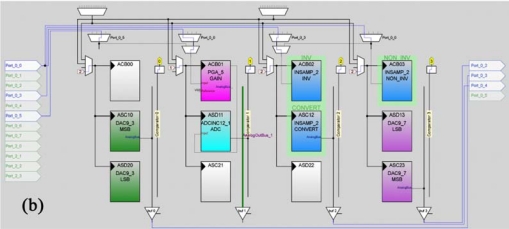
Resistive Sensors: **(a)** Logical scheme of UISI conditioning front-end and **(b)** screen-shot of related implementation in PSoC device through by means of Graphic Configurator.

The conditioning of the resistive sensor is the most complex case [see Figure 8(a)]. Power is not directly supplied by a buffered DAC as in the other cases. In this configuration, the resistor is supplied by a constant current generator. In order to achieve this goal, a particular configuration involving a DAC module, a differential and unitary gain amplifier, and an external precision resistor (R_0_) is applied (the resistor is external to the PSoC but is included in the complete UISI and cannot be changed by configuration parameters).

The reference current obtained with this configuration (the value of reference current is I_0_ = −V_0_/R_0_, where V_0_ is fixed by DAC) flows across the resistive sensor. In this way, the resistance is converted at the resistive sensor terminals into a voltage. This voltage is amplified by a differential amplification stage: the gain is automatically selected on the basis of the configuration parameters loaded in the TEDS. The last module is the ADC (with 12 bit resolution).

Figure 8(b) shows the implementation of the analog part of this configuration, carried out using Graphic Configurator. In this configuration no supply voltage directly connected to the sensor is present. The DAC9_3 supplies the reference voltage to the circuit in order to fix (together with the R_0_) the current I_0_ that flows in the resistive sensor. The reference voltage is applied to the negative input of the INSAMP_2, set with a gain of 1. The non-inverted input is connected to the R_0_ and the Sensor by the PORT_0_0. Moreover, it is connected with the PGA_5 that amplifies the signal finally acquired by the ADCINC12_1. The PORT_0_4 (R_0_′) is connected to the ground via firmware. DAC9_7 is the DAC for the analog output:

(4) $$V_{\mathrm{ADC}}=\left(-I_{0}\times R_{S}\times G\right)=V_{0}\times R_{S}\times G/R_{0}$$

#### 3.2.5. Capacitive Sensor—Configuration 5

**Figure 9.**
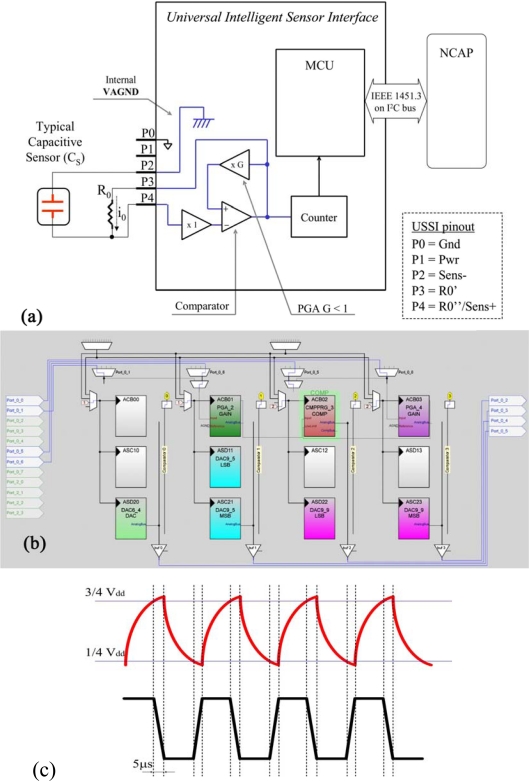
Capacitive sensors: **(a)** Logical scheme of UISI conditioning front-end and **(b)** screen-shot of related implementation in PSoC device through by means of Graphic Configurator. **(c)** Signal on comparator (-) input (top) and signal on output (bottom).

The conditioning of the capacitive sensor was achieved by the use, also in this case, of an external precision resistor. The resistor is external to the PSoC, but it is included in the UISI device, and cannot be changed by configuration parameters. Figure 9(a) shows the conditioning scheme for this configuration. The internal circuit, together with the external resistor and the capacitive sensor, realizes an oscillating circuit with time constant:

(5) $$\tau =1/R_{0}\times C_{S}$$

The thresholds for the commutation of the oscillator are fixed by the amplifier with amplification G, with G less than 1. In particular, with a Gain of 0.5, the thresholds of the comparator are set to: ¼ V_DD_ and ¾ V_DD_ (see Figure 9(c). In this condition the period of oscillation of the circuit is:

(6) $$T=2\times R_{0}\times C_{S}\times \text{ln}\;3$$

The output of the oscillator is sent to a counter that counts the number of pulses in a period and computes the frequency of the signal. For the circuit to function correctly the frequency of oscillation must be kept under 40 KHz. Furthermore, a correction factor was introduced to take into account of the slow rate of the comparator that introduce a delay due to the high-to-low and low-to-high commutations at the output of the comparator. Each commutation introduces a 5 μs delay, for a total of 10 μs in a period [see Figure 9(c)]. Consequently, in the calculation of the period (and frequency) a correction must introduced:

(7) $$T_{C}=T_{M}-0.00001\;\;;T=1/f\;\;\Rightarrow \;\;f_{C}=1/\left(1/f_{M}-0.00001\right)$$

being *T_M_* and *f_M_* the period and the frequency measured by the circuit, respectively, and *T_C_* and *f_C_* the period and the frequency calculated with the correction factor.

Figure 9(b) shows the implementation of the analog part of this configuration, carried out using the Graphic Configurator. As in the configuration 1, the first block is a DAC6 used for generating the power supply of the sensor. The sensor output is connected to the PORT_0_0 (Sens+) and to the PORT_0_5 (Sens−) that is internally connected to AGND by the DAC9_5. The core of the configuration is the CMPPRG_3 that has the input connected to the PGA_2. The PGA_2 has a fixed gain <1 set during the configuration and receives the comparator output signal. The PGA_2 output is then connected as an input to the comparator itself. The second amplifier, PGA_4, is used as an analog input buffer from the capacitive to the input of the comparator. The output of the comparator is sent also to a counter that measures the frequency of the signal. DAC9_9 is the DAC for the analog output.

#### 3.2.6. Digital Sensor—Configurations 6, 7 and 8

**Figure 10.**
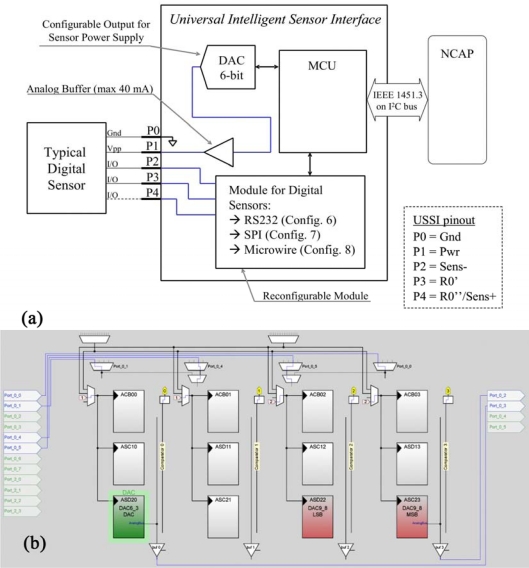
Digital sensors: **(a)** Logical scheme of UISI conditioning front-end and **(b)** screen-shot of related implementation in PSoC device through by means of Graphic Configurator.

Digital sensors represent a large share of used commercial sensors. The UISI device is potentially able to interface with most of them. In any case, the huge number of different protocols and communication interfaces make the proposal of a single generic configuration able to cover the entire range of sensors unrealistic. In this case *ad-hoc* solutions are necessary for each device. For this reason configurations for digital sensors were designed, but not fully implemented. Figure 10 shows the scheme related to the implementation of digital sensor configurations.

Three main hardware communication interfaces have been selected as representative of different digital sensors, and implemented at the hardware level: RS232 serial communication (corresponding to configuration 6), SPI (Serial Peripheral Interface) [33] (corresponding to configuration 7) and I^2^C [34] (corresponding to configuration 8). All these protocols are supported by the PSoC device at the hardware level with working parameters completely configurable at run-time. Nevertheless, the firmware protocol has to be developed *ad hoc* for each sensor. Obviously digital sensors do not require the configuration of analog blocks (except, optionally, for the sensor power supply).

### 3.3. Firmware Design

#### 3.3.1. IEEE 1451.3 over I^2^C Implementation

The 1451.3 standard contemplates a connection between the NCAP (Network Capable Application Processor—defined in the IEEE 1451 standard) and the intelligent sensors (UISIs) through a specific bus. In our application we adopted a different physical layer based on the I^2^C bus and a reduced implementation of the protocol. It uses in addition to the standard I^2^C lines (Data and Clock), two more digital lines:

- One Detection line, used for starting the *plug&play* service when a new UISI is connected to the NCAP;
- One Trigger line, used to wake up the UISIs before the sending of a command on the I^2^C bus.

We used a customized protocol and not a fully compliant 1451.3 protocol mainly because the requirements for implementing the physical layer and other software layers are too demanding to be implemented on a relatively simple and low power consumption device, such the one we proposed.

#### 3.3.2. Firmware Block Diagram

**Figure 11.**
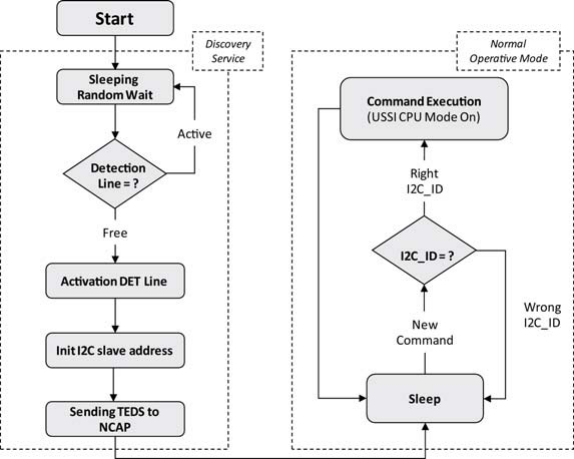
Firmware block diagram.

The UISI device has to guarantee several operational functionalities to the sensor. The firmware for the management of the UISI is divided into two functional blocks:

- Firmware for the management of the hardware configuration;
- Firmware for *plug&play* and data communication;

The firmware implementation is described in Figure 11. At Power On Reset (POR) or when an UISI is connected (or reconnected) to the bus, a procedure for the I2C_ID assignment and TEDS transmission (required operation for “Plug and Play” connectivity) is activated. The assigned I2C_ID has to be stored in a volatile memory (reset at power on) and compared at each following I^2^C communication with the ID sent by NCAP. After the initialization phase, the UISI runs in Low Power Mode (CPU Sleep), with all power line of the Transducer channels switched off. The UISI must remain in sleep state until the detection of a communication frame over the I^2^C bus: at this point the UISI wakes-up from the Sleep mode, decodes the I2C_ID and, if the I^2^C address read on the line matches with its own I2C_ID, executes the received command. After the command execution the UISI set back to Sleep mode. If the address is not a matching address, UISI set back to Sleep mode immediately, without waiting the end of I^2^C frame transmission.

Once the UISI is woken up, the firmware starts the sequence for PSoC hardware configuration, consisting in a series of functions setting-up the front-end for the connected sensor. In particular such functions are:

- TedsRead(): reads from the TEDS the type of sensor connected to the UISI (capacitive, resistive, unconditioned…)
- ConfigLoad(): loads the proper analogical configuration for sensor conditioning
- FrontEndOn(): switches on the required blocks and selects the correct parameters (gain, supply voltage, *etc.*) on the base of information read from TEDS
- SensorWarmUp(): warms-up the sensor as specified by the proper parameter (from 100 ms to 25.6 s).
- ReadSensor(): acquires the sensor signal
- FrontEndOff(): switches off all the modules
- Sleep(): puts the device in Sleep mode.

The data communication is based on a customized subset of commands defined by the IEEE 1451 standard, guaranteeing the complete control of the UISI by the NCAP (see the protocol section for a complete command list). Basically the NCAP communicates with the UISI through the customized I^2^C bus hardware; NCAP is the master and the UISIs are the slaves. The NCAP sends a frame (a Command that could contain data) to the UISIs that answer with another frame (that could contain also data). The selection of the right UISI is made by the I2C_ID. For this reason at the first connection with the NCAP a unique I2C_ID address is assigned to each UISI.

When a new UISI is connected to the NCAP, a procedure for the plug & play of the new intelligent sensor begins. This procedure is called Discovery Service and causes the following results:

- the assignment of the I^2^C address;
- the presentation of the sensor to the Network through the transfer of the related TEDS.

In order to activate the Discovery Service, the connected UISI must activate the detection line (if it is not already activated). To guarantee the plug & play capabilities when a stack of UISI is powered on (or connected to the bus at the same time), a random delay time has been introduced at the UISI power up. In this way the probability that two or more UISIs enable the detection line at the same time is very low (lower than 10^−5^); anyway, if a collision occurs the system get back an error during the I2C_ID assignment and the procedure (with random delay) is started again. This assures that no UISIs are lost during the plug & play procedure.

#### 3.3.3. Considerations on Power Consumption

One of the main issues in the design of the UISI and in the development of the firmware is the power consumption. The capability to operate in a power safe mode is important given that these systems are designed for continuous operation; low power consumption has the advantage of permitting the reduction of the power supply device (e.g., the surface of a solar panel and battery dimensions). The UISI has three main operational states, characterized by different power consumptions:

- Sleep Mode: the PSoC CPU is in sleep mode and all the analog and digital blocks are turned off, the UISI is waiting for a new command;
- Elaboration Mode: the PSoC CPU runs at 3 MHz and all the analog and digital blocks are turned off; the UISI is processing a command and/or preparing output data for the NCAP;
- Measuring Mode: the PSoC CPU runs at 3 MHz and the configured analog and digital blocks are turned on; the UISI is performing a new measurement;

The selection of 3 MHz frequency for the CPU clock is a compromise between different aspects. A very high clock speed is not necessary in this application because most of the time is spent in the transmission of data through the I^2^C bus. On the other hand, a too slow clock does not permit a correct operation of the I^2^C module and increases the wake up time from the Sleep Mode. With this solution the following performances in term of power consumption have been obtained (@ 5 V): 3.9 μA in Sleep Mode; 5.1 mA in Elaboration Mode (only CPU); 9.9 mA in Measuring Mode (with analog and digital blocks on, considering the worst configuration in terms of consumption: 2 DAC9, 1 DAC6, 2 PGA, 1 ADC, 1 Timer and 1 Counter). The UISI Wake up time is 200 μs.

Because of the fact that all the UISIs are woken up at the same time from a NCAP trigger event, a control on the I^2^C address has been inserted at frame receiving time; in this way the UISIs not interested by the command can immediately return in Sleep Mode after the complete reception of the address (in less than < 500 μs). Average power consumption depends on sampling rate and sensor warm-up time. In typical sensor network applications with sampling rate of 0.1 sample/s, and 0.5 s of sensor warm-up time, the overall UISI average current consumption is in the order of 400 μA (detailed calculation are reported in Table 1) and thus the power consumption is in the order of 2 mW. With shorter warm-up times smaller power consumptions are achieved (more than one order of magnitude smaller).

### 3.4. Design Limitations, Absolute Maximum Ratings and Expected Accuracy

The presented UISI device design guarantees a series of advantages in terms of flexibility, scalability, costs and ease of implementation. Nevertheless the conditioning capability and achievable performance are strictly dependent on the architectural choices. In fact, due to the available PSoC resources, the sensor to be conditioned must respect some limitations in terms of power requirements and working ranges. The absolute maximum ratings for each type of sensor (summarised in Table 2) are discussed in the following.

Configuration n° 1 does not present major limitations, except for the 0–5 V input range. Configuration n° 2 presents a first limitation in the used architecture: the maximum differential output signal of the sensor cannot be higher than 2.5 V due to the first stage of amplification that cannot have a gain lower than 2. This limit is reachable only with null offset. However, the signal is usually very low for the differential output sensors consequently this is not a severe limitation. Theoretically no minimum input should be fixed, anyway by decreasing the measurement dynamics there is a loss of resolution. In fact, the maximum amplification with this configuration is 16 × 48 = 768; with a signal lower than 6.5 mV the whole dynamics of the ADC cannot be exploited. A limitation on the input common mode signal is present to operate in the optimal working range of the instrumentation amplifier. Moreover, the maximum permitted offset depends on the gain of the first amplification stage and on the maximum differential signal; in many cases it should be maintained under 150 mV.

Regarding Configuration n° 3, no limitations are specified for the highest measurable current, because the input range is regulated by the chosen external resistor R_0_. The smallest measurable current, instead, is limited to 10 μA by the PSoC Input Leakage Current (that is in the order of 0.2 uA). Differently, for resistive sensor configuration (Configuration n° 4) there is a range of resistance that must be respected to guarantee a good performance of the measurements. In particular the maximum resistance must be lower than 2 MΩ in order not to interfere with the internal resistances of the PSoC. The minimum measured range must be kept above the 0–50 Ω in order to avoid a loss of resolution. For the range of capacitances measurable with Configuration n° 5, no major restrictions are present. The only restrictions concern the minimum capacitance that has to be above 200 pF in order not to interfere with the PSoC’s own internal capacitances. Another limitation regards the minimum capacitance that can be measured once that the maximum one has been fixed. This configuration measures the capacitance by the indirect measurement of the frequency; in order to have at least a resolution of 1% for the maximum sensor capacitance, an oscillation of at least 100 pulses in the sampling time (fixed to 500 ms) must be guaranteed; in these conditions the measured signal has a frequency of 200 Hz. By using this circuit the maximum measurable frequency is 40 KHz. Being the frequency inversely proportional to the signal period, the minimum capacitance measurable is 200 times lower than the maximum one. This methodology presents a resolution that is not constant in the whole range of measurable capacitances: but the resolution is high with low capacitances to measure and decreases when large capacitances are present. Sensor constrains and absolute maximum rating for UISI interfaces.

Regarding the accuracy achievable by the devices, it clearly depends on the selected configuration and operative conditions. The main sources of errors from analog measurements arise from the ADC and PGA modules. Each module has an offset error (here reported as ΔErr_ADC_ and ΔErr_PGA_) and a gain error (here reported as %Err_ADC_ and %Err_PGA_). The absolute value of these errors strongly depends from temperature; moreover, the %Err_PGA_ error depends also from the selected amplifier gain (%Err_PGA_ is minimum for G = 1). Considering these error sources it is possible estimate the accuracy for each configuration.

Let consider for example the simple Configuration 1: the input signal is collected through the PGA stage (with unitary gain) and then converted by the ADC, than measured potential V_meas_ is thus affected by the offset and gain errors as follows:

(8) $$V_{\mathrm{meas}}=\left(V_{\mathrm{in}}\cdot G\cdot \left(1+\%{\mathrm{Err}}_{\mathrm{PGA}}/100\right)+\Delta {\mathrm{Err}}_{\mathrm{PGA}}\right)\cdot \left(1+\%{\mathrm{Err}}_{\mathrm{ADC}}/100\right)+\Delta {\mathrm{Err}}_{\mathrm{ADC}}$$

where is G = 1.

By using the nominal error values reported in PSoC datasheets it is possible to estimate the accuracy of the devices. In particular, for Configuration 1, at environmental temperature (25 °C) a nominal error of 0.9% has been calculated (ΔErr_ADC_ = 9 mV, ΔErr_PGA_ = 4,5 mV, %Err_ADC_ = 0,1%, %Err_PGA_ = 0,5%). The error increases substantially if we consider the whole temperature range (from −40 °C to 80 °C): in this case the error is the order of 2,4% considering typical ADC and PGA error values (ΔErr_ADC_ = 12 mV, ΔErr_PGA_ = 14 mV, %Err_ADC_ = 0.5%, %Err_PGA_ = 1.4%).

## 4. Experimental Setup

**Figure 13.**
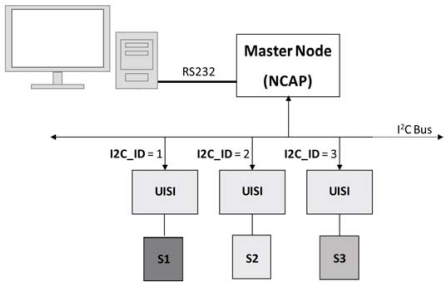
Experimental setup scheme.

In order to test the UISI functionalities, a Master Node performing the functions of NCAP was implemented. This node communicates with a PC by a RS232 interface and with the UISIs by the I^2^C custom bus. In particular the NCAP automatically manages the UISI’s plug & play connections and assigns the I^2^C addresses. Then, controlling NCAP by using an *ad hoc* Software User Interface, it is possible to send all the implemented commands to the specific UISI (Figure 13). In this configuration, it is possible to simultaneously manage up to16 UISIs.

**Figure 14.**
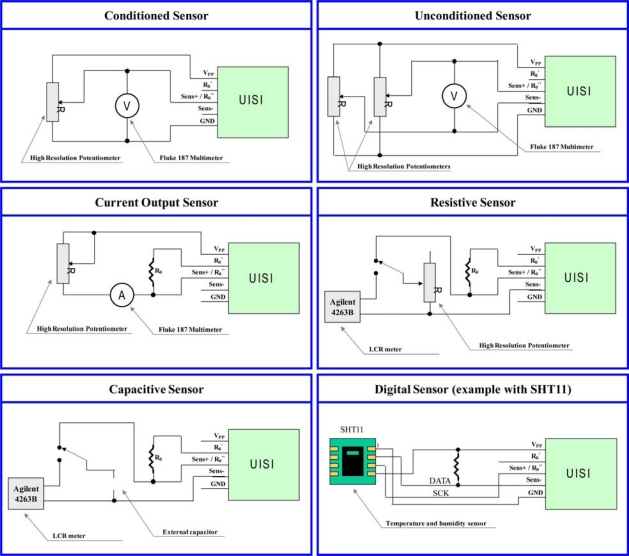
Configuration setups for the proposed sensor configuration front ends.

The testing of the conditioning configurations of UISI was carried out with different experimental set ups. To make precise reference measurements two precision instruments were employed: a precision *True RMS Multimeter* (Fluke 187) for the measurements of voltage and current; a *LCR Meter* (Agilent 4263B) for the measurements of resistance and capacity. Figure 14 shows in details the used configuration set-ups.

## 5. Results and Discussion

### 5.1. Test Results

The UISI was tested both for the protocol and for the electrical performance in the different proposed configurations. Several tests were performed to check the protocol with the NCAP simulator connected to the PC; all the commands developed were successfully tested and a fully reliable communication is assured up to 16 UISIs simultaneously connected.

Concerning the electrical performance of the UISI device, each configuration will be separately discussed in the next sections considering the precision, resolution, linearity and sensitivity. A summary of these characterization results is finally reported on Table 3.

#### 5.1.1. Conditioned Sensor Tests

**Figure 15.**
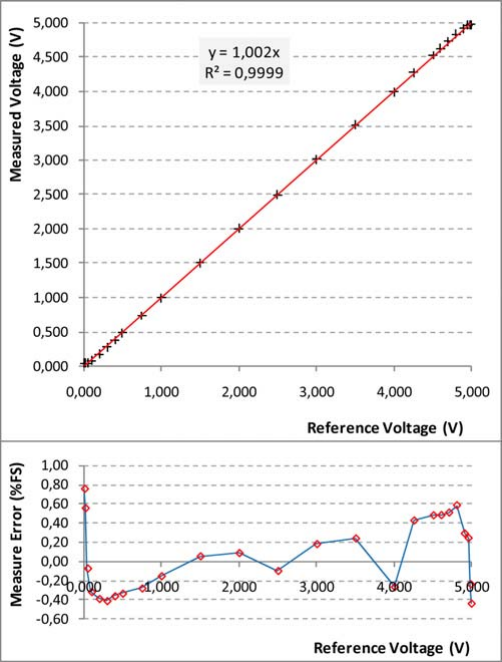
Results of characterization test carried out on UISI Conditioned Sensor configuration. Top graph show the measured voltage *vs.* the reference voltage; Bottom graph shows the corresponding error in Full Scale (FS) percentage.

The “Conditioned Sensor” configuration is the simplest one to test. Only a potentiometer and a multimeter are used in the characterization of this configuration (see Figure 14). The results of the test are shown in Figure 15. The configuration shows a high precision except for input signal lower than 50 mV and greater than 4.95 V where a saturation behavior is present. The resulting maximum error is 0.75% of the full-span scale (FS) in proximity of the extremes. In the central zone of the input span the maximum error results usually less than 0.2% FS.

#### 5.1.2. Unconditioned Sensor tests

**Figure 16.**
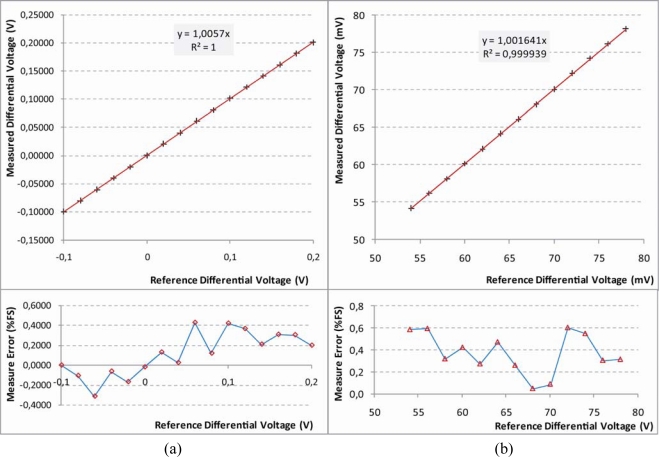
Results of characterization test carried out on UISI Unconditioned Sensor configuration for **(a)** high differential signal with small offset and **(b)** small differential signal with high offset. In both figures the top graph shows the measured voltage *vs.* the reference voltage; bottom graph shows the corresponding error in Full Scale (FS) percentage.

This is the most critical configuration since a very accurate set up of the internal hardware must be performed before the utilization. To test this, several different conditions were simulated. As an example, we report here in detail the results obtained for two tests:

- The first test with an input signal ranging from −0.1 V to +0.2 V. The configuration parameters set to condition the input signal are: 1^st^ stage gain = 8; V_ref_ = 2.1 V; 2^nd^ stage gain = 1. The results are shown in Figure 16(a). The maximum error results to be lower than 0.4% FS.
- The second test with an input signal ranging from 0.054 V to 0.078 V. This signal presents a high offset (compared to the magnitude of the signal). For this configuration the following parameters were fixed: 1^st^ stage gain = 16; V_ref_ = 1.46 V; 2^nd^ stage gain = 8. The results are shown in Figure 16(b). The maximum error was less than 0.6% FS.

#### 5.1.3. Current Output Sensor Tests

**Figure 17.**
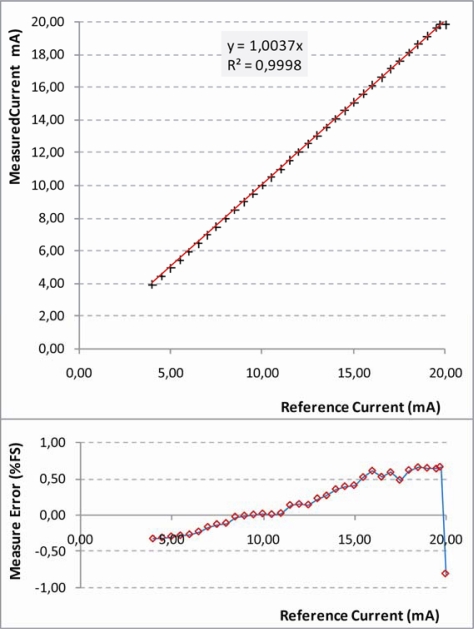
Results of characterization test carried out on UISI Current Output Sensor configuration. Top graph show the measured current *vs.* the reference current; Bottom graph shows the corresponding error in Full Scale (FS) percentage.

In this configuration a potentiometer and a multimeter are used (see Figure 14). An external high precision resistor is also necessary. The configuration was tested for a typical current output of 4–20 mA, being a standard output for many commercial sensors. For this current range the external resistor was chosen to be 250 Ω. The results are shown in Figure 17.

In this case the saturation behavior happens only near the upper limit (5 V), due to the range of the input current, resulting in a maximum error of −0.81% FS. Similar results can be obtained with lower current value and higher external resistor.

#### 5.1.4. Resistive Sensor Tests

**Figure 18.**
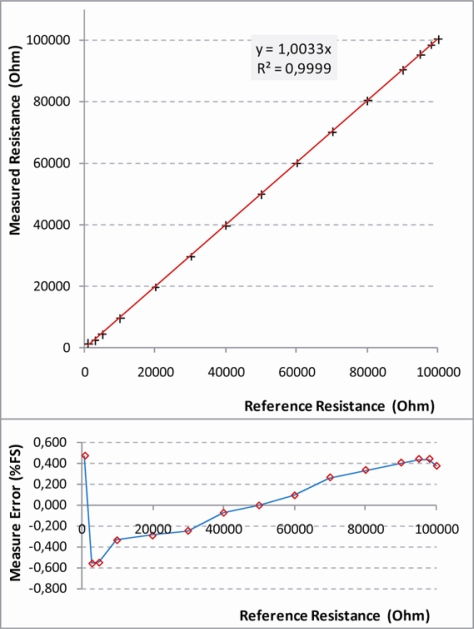
Results of characterization test carried out on UISI Resistive Sensor configuration. Top graph show the measured resistance *vs.* the reference resistance; Bottom graph shows the corresponding error in Full Scale (FS) percentage.

In this configuration an external precision resistor is necessary. The resistance range that can be correctly measured is selected on the base of the value of external reference resistor (R_0_) and of two internal UISI parameters (Gain and V_ref_) The configuration was tested in the range from 1 KΩ to 100 KΩ by using a series of high precision resistances. The following parameters were fixed for the characterization: Gain = 1.6; V_ref_ = 1 V; R_0_ = 50 KΩ. The results are shown in Figure 18.

These results show a quite good accuracy in the measurements of values of the input resistance between 1 KΩ and 100 KΩ where the maximum error is less than 0.5% FS. With a resistance greater than 100 KΩ a saturation behavior was observed because the voltage is near 5 V. A similar behavior (saturation near 0 V) is present for resistances lower than 1 KΩ. For this reason, with this configuration it is possible to measure with a good precision a resistance that varies no more than two orders of magnitude.

#### 5.1.5. Capacitive Sensor Tests

**Figure 19.**
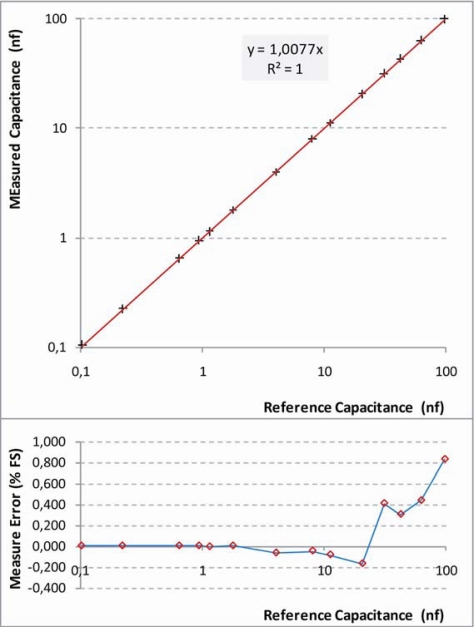
Results of characterization test carried out on UISI Capacitive Sensor configuration. Top graph show the measured capacitance *vs.* the reference capacitance; Bottom graph shows the corresponding error in Full Scale (FS) percentage.

The configuration for the measurement of capacitive sensor requires the use of an external resistor. The capacitance range that can be correctly measured is selected on the base of the value of external reference resistor (R_0_). The configuration was tested in the range from 0.1 nF to 100 nF, fixing the following parameters: R_0_ = 100 KΩ; Sampling Time = 0.5 s. The results are shown in Figure 19. For small capacitances the frequency of oscillation is high. This results in a high resolution and consequently in a small measurement error. The relative error is lower than 0.2% FS for most of the capacitance range. Starting from capacitances two orders of magnitude larger than the smallest one the error increases with the increment of the capacitance reaching 0.8% FS for a capacitance three orders of magnitude greater than the smallest one.

### 5.2. Sensors Integration—Application Examples

In addition to standard lab bench tests (just reported), the UISIs were further tested and optimized by intensive use in several real application fields.

#### 5.2.1. UISI for WSN

**Figure 20.**
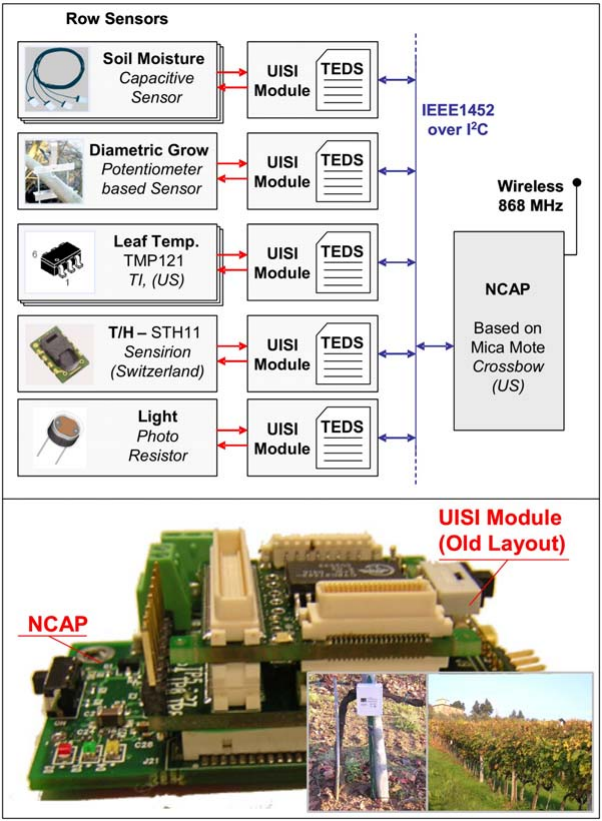
Examples of sensors integrated in a vineyard and a picture of the system.

The first application of the UISI was in monitoring physical parameters in the agricultural field [35]. Different sensors were integrated in a wireless sensor network in a vineyard. The UISI allowed the creation of an open infrastructure with the possibility to add new sensors at any time and to exchange the sensors between the wireless nodes. In Figure 20 some examples of sensors integrated by the UISI in the vineyard are shown along with an overview of the system implemented in field.

#### 5.2.2. UISI for Pollution Monitoring

**Figure 21.**
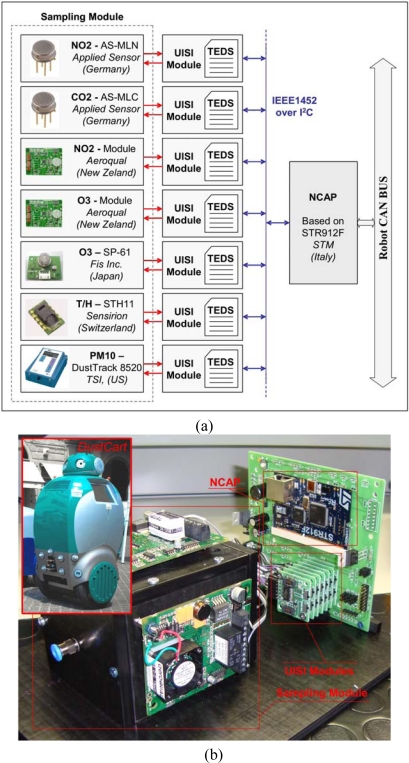
**(a)** Examples of sensors integrated in the mobile robots; **(b)** pictures of the system.

The UISI was also used in a mobile robot application to map the distribution of pollution in urban environments [36,37]. In this case the used sensors were mainly devoted to gas pollution monitoring (see Figure 21). In this application the characteristics of the UISI solution were shown to be important both for the possibility of frequently replacing the gas sensors for calibration (a calibration curves feature is included in the UISI) and for the possibility to integrate at any time new sensors without the need to modify the acquisition module.

The same concept is also used in several other applications (e.g., in the automotive field for integrating hydrogen sensors or in geotechnical application to integrate sensors for landslip monitoring).

## 6. Conclusions

This paper presents the concept of a Universal Intelligent Sensor Interface, a new low-cost system developed by the authors, which is able to convert a generic transducer into an intelligent sensor with multiple standardized interfaces. The general concept was introduced and a functional design based on firmware reconfigurable hardware was proposed.

The UISI device can implement up to eight different analog and digital front-ends to interface several types of sensor outputs, including: conditioned and unconditioned (Wheatstone bridge like) sensors, capacitive and resistive transducers, current sensors and digital sensors. Plug and play functionality based on the concept of IEEE 1451 Transducer Electronic Datasheets is also supported.

All the important aspects related to the realization of the UISI technology have been discussed, including system architecture, hardware and firmware design, realization and validation. The architectural design of all the proposed configurations was analysed in detail and the achievable performances and limits were evaluated, also in terms of power consumption.

An accurate series of lab bench tests have been carried out to characterize the UISI devices and the real performances of the integrated system (in term of accuracy, off-set, resolution, linearity and ranges) showing, in general, characteristics compatible with the most common monitoring applications (e.g., total error lower than 1% FS in all the configurations and supported ranges). Finally a couple of real world application examples have been also provided.

The UISI device represents an innovative solution to face the growing needs to integrate intelligent sensors in real time, reliable, low maintenance distributed monitoring systems and wireless sensor network systems, which have been becoming more and more present in several applicative fields, such as environmental, agro-alimentary, medical and industrial ones.

## Figures and Tables

**Table 1.** Detailed UISI electrical power calculations.

**Operative Status (One Sample)**	**Time (ms)**	**Current @ 5 V (μA)**	**Energy @ 5 V (μAh)**
Sleep	9340	3.9	0.01
Sensor warm up time	500	5100	0.71
Sensor Acquisition	150	9900	0.41
Other processor activities	10	5100	0.014

One Sample Consumption (μAh)			1.15
Average Consumption (μA)		412.2	

**Table 2.** Detailed UISI electrical power calculations.

All Configurations	
- Maximum Sensor Power Supply Voltage	5 V
- Minimum Sensor Power Supply Voltage	1 V
- Maximum Sensor Power Supply Current	30 mA

Conditioned Sensor – Conf.1	
- Max Conditioned Sensor Output	5 V

Unconditioned Sensor (Wheatstone bridge like) – Conf.2	
- Max Unconditioned Sensor Output (common mode)	4 V
- Min Unconditioned Sensor Output (common mode)	1 V
- Max Unconditioned Sensor Full Scale Output (differential)	2.5 V
- Min Unconditioned Sensor Full Scale Output (differential)	10 mV
- Max Unconditioned Sensor Offset Output (differential)	150 mV

Current Output Sensor – Conf.3	
- Max Current Sensor Output	n.a.*
- Min Current Sensor Full Scale Output	10 μA

Resistive Sensor – Conf.4	
- Max Full Scale Resistance	2 MΩ
- Min Full Scale Resistance	50 Ω

Capacitive Sensor – Conf.5 (16 bit of resolution – error < 2%)	
- Max Full Scale Capacitance	n.a.*
- Min Full Scale Capacitance	100 pF

Digital Sensor – Conf.6/7/8	
- Logic I/O	3.3–5 V
	compliant

* It depends from the value of the additional external resistor.

**Table 3.** Summary of UISI characterization results for the different sensor configurations.

**UISI Configuration**	**Accuracy (Max Error)**	**Off-Set**	**Resolution**	**Nonlinearity**	**Sensitivity Error (%)**	**Tested Range**
Conditioned Sensor	0.8% FS	5 mV	1.2 mV	0.8% FS	0.2%	0 ÷ 5 V
Unconditioned Sensor (G x8)	0.4% FS	100 μV	150 μV	0.3% FS	0.6%	−0.1 ÷ 0.2 V
Unconditioned Sensor (G x128)	0.6% FS	200 μV	10 μV	0.5% FS	0.2%	54 ÷ 78 mV
Current Output Sensor (4–20 mA)	0.7% FS	0.1 mA	5 μA	0.2%FS	0.4%	4 ÷ 20 mA
Current Output Sensor (Test)	0.7% FS	0.4 μA	25 nA	0.3% FS	0.3%	5 ÷ 100 μA
Resistive Sensor	0.6% FS	300 Ω	25 Ω	0.2% FS	0.3%	1 ÷ 100 KΩ
Capacitive Sensor	0.8% FS	0.06 nF	2E-6 ÷ 2 nF	0.3% FS	0.8%	0.1 ÷ 100 nF
